# Shear bond strength of rebonded metal brackets using four light-curing resins: an in vitro study

**DOI:** 10.2340/biid.v13.45703

**Published:** 2026-04-16

**Authors:** Verónica Estefanía Pozo, Mauricio Aguirre Balseca, Marjory Elizabeth Vaca Zapata, Karina Maria Salvatore Freitas, Johanna Elizabeth Fiallos Sanchez

**Affiliations:** a Department of Health Sciences, University of the Hemispheres, Quito, Ecuador; b Ingá University Center UNINGA, Maringá, Brazil

**Keywords:** bracket adhesion failure, bracket reconditioning, light-cure orthodontic adhesives, orthodontic rebonding, sandblasting surface treatment

## Abstract

**Background:**

Rebonding of orthodontic brackets requires effective surface conditioning to restore adequate adhesive retention.

**Objective:**

To compare the shear bond strength (SBS) of rebonded metal brackets using four light-curing resins.

**Material and methods:**

Eighty premolars were allocated to two conditioning protocols (*n* = 40 per group): G1 – enamel etching with 37% phosphoric acid; G2 – sandblasting of bracket bases followed by enamel etching. Each group was subdivided according to the resin used (*n* = 10 per subgroup): Orthocem (FGM), Bracespace (3M), Transbond XT (3M), and Z250 (3M). Brackets were bonded, debonded, reconditioned, and rebonded. SBS was tested using a universal testing machine (0.5 mm/min). Data were analyzed using Kruskal–Wallis and Dunn’s post-hoc tests (α = 0.05).

**Results:**

In the acid‐etched group, there was no statistically significant difference in SBS among the four resins (Kruskal–Wallis, *p* = 0.097). In the sandblasting group, a significant intergroup difference was observed (*p* = 0.044). Dunn’s post-hoc test identified higher SBS for Bracespace (3M) and Transbond XT (3M) compared with Orthocem (FGM) and Z250 (3M). When comparing conditioning methods within each resin, sandblasting of the bracket bases resulted in significantly higher SBS only for Bracespace and Transbond XT, whereas Orthocem and Z250 showed no significant change.

**Conclusions:**

Rebonding of metal brackets showed comparable SBS among resins after acid etching. However, when the bracket bases were sandblasted, Bracespace and Transbond exhibited significantly higher bond strengths. Surface preparation of the bracket base is therefore a key factor influencing SBS in rebonding procedures.

## Introduction

Bracket bonding is a fundamental procedure in orthodontics, requiring precise control over the adhesion process to ensure long-term treatment success. This process relies on the unique properties of dental enamel, which forms the outermost, hardest tissue of the tooth. Enamel is composed primarily of tightly packed hydroxyapatite crystals (approximately 95% by weight), with smaller fractions of organic matter (1–2%) and water (2–4%) [1, 2]. Although exceptionally strong, mature enamel is a non-regenerative tissue, making its preservation a critical aspect of orthodontic care [3].

Orthodontic bonding has evolved significantly since Edward Hartley Angle introduced the first Edgewise brackets in 1928, which were initially bonded using bulky metal bands. The development of acid etching by Michael Buonocore in the 1950s revolutionized orthodontic bonding, enabling direct attachment of metal, ceramic, and plastic devices to enamel surfaces [4]. This innovation reduced the need for metal bands, improving esthetics and patient comfort. Despite these advances, bracket detachment remains a common clinical issue, disrupting treatment progress and increasing chair time [5, 6].

Adhesion in orthodontics involves the attraction between two surfaces at the molecular level, relying on the interaction between the adhesive resin and the roughened enamel surface. Proper bonding requires the creation of a strong mechanical interlock between the composite resin and the bracket base, which is facilitated by increasing the surface energy through techniques like acid etching and sandblasting [7]. However, the strength of this bond can be compromised by factors such as enamel contamination, inadequate surface preparation, and improper adhesive selection [8, 9].

Shear forces are particularly critical in orthodontic bonding, as they directly influence the stability and durability of the bracket-enamel interface. These forces act parallel to the long axis of the tooth, testing the strength of the adhesive bond during masticatory loading [10, 11]. If the bond is too weak, brackets may detach prematurely, extending treatment time and reducing patient satisfaction [12, 13].

Bracket rebonding, often necessary after bond failure, presents its own challenges. The process can damage enamel, cause tooth collisions, and lead to gingival recession if not properly executed [14]. Effective rebonding depends on thorough cleaning of the bracket base, removal of residual adhesive, and proper surface conditioning to restore bond strength [15]. Techniques like sandblasting can significantly enhance bond strength, creating a micro-retentive surface that significantly improves adhesive retention [16]. In line with this evidence, Dirie et al. [17] demonstrated that sandblasting combined with acid etching yields significantly higher bond strengths during rebonding compared to acid etching alone, reinforcing the importance of adequate enamel pretreatment before bracket replacement.

However, few studies have compared the performance of these specific adhesive systems during rebonding procedures, and there is still insufficient evidence to determine whether different resins respond differently to base surface conditioning.

Given the critical importance of achieving and maintaining strong adhesive bonds in orthodontics, this study aims to evaluate the shear bond strength (SBS) of rebonded brackets using different light-curing resins. The findings will contribute to a better understanding of the factors that influence long-term bracket stability and treatment success. The hypothesis to be tested is that sandblasting of bracket bases prior to rebonding increases SBS compared with acid etching alone, and that this improvement may vary among the different adhesive systems evaluated.

In this study, four resins were selected because they are widely used clinically in our region and represent distinct formulations with differing viscosities, filler loads, and handling characteristics, thus allowing a meaningful comparison of their performance under identical bonding and rebonding conditions.

## Material and methods

This study was approved by the Institutional Ethics Committee of the University of the Hemispheres, Quito, Ecuador, under protocol number CEUHE25-48 (IRB reference). All procedures followed the principles of the Declaration of Helsinki for biological material handling. Written consent for tooth donation was obtained under institutional Tooth Donation Release Form, and all teeth were anonymized before testing. This study was an experimental, in vitro, descriptive-comparative investigation designed to evaluate the SBS of rebonded brackets using different light-curing resins.

All extracted premolars were obtained from patients who provided written informed consent allowing the use of their teeth for research purposes. Teeth were collected through the clinic’s standard biowaste donation protocol and handled according to institutional and bioethical regulations.

### Sample collection and preparation

The sample size of *n* = 10 specimens per subgroup was adopted based on methodological standards commonly reported in in-vitro SBS studies. Systematic reviews indicate that SBS experiments typically use 8–12 samples per group, due to laboratory feasibility constraints and the destructive nature of the test design [18, 19]. Therefore, *n* = 10 was selected as an appropriate and widely accepted sample size for this type of analysis.

Extracted premolars were obtained according to the institutional tooth donation consent protocol, logged by receipt codes T01–T80 in the university tooth biobank registry.

The sample consisted of 80 extracted premolar teeth (both maxillary and mandibular), obtained over a 1-year period from orthodontic patients at the Ángel Dental Center in Quito. Teeth were extracted for orthodontic reasons and collected in accordance with ethical guidelines. Following extraction, blood and remaining soft tissue were removed using conventional water irrigation and a Maillefer 51–52 curette. The teeth were then stored in physiological saline solution at room temperature (10–25°C) to preserve their integrity until testing.

**Figure 1:**
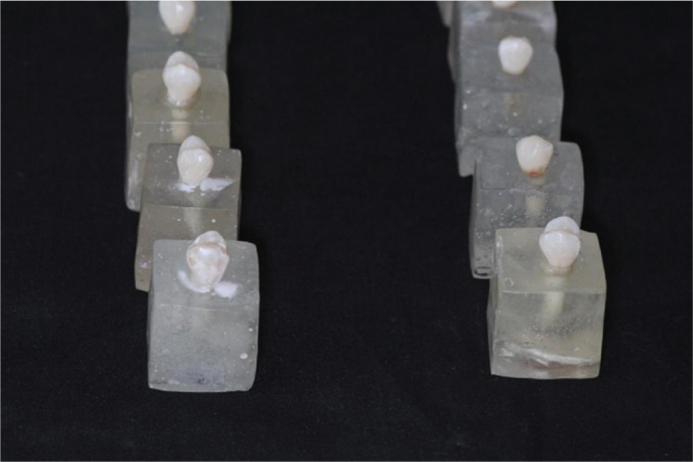
Premolars mounted in acrylic blocks.

The premolars were mounted in acrylic blocks measuring 2 cm in height and 2 cm in width, leaving the crowns exposed for bonding (Figure 1).

Similar storage and acrylic embedding techniques have been widely applied in previous in-vitro SBS studies, confirming their appropriateness and reproducibility for this type of experimental setup [20].

### Inclusion and exclusion criteria

Inclusion Criteria:

- Intact, healthy crowns without caries or previous orthodontic treatment.
- No history of endodontic treatment.

Exclusion Criteria:

- Teeth with fluorosis, malformations, or crown fractures.

Elimination Criteria:

- Brackets that detached prematurely before shear testing.
- Premolars that fractured during the shear bond tests.

### Randomization and blinding

The 80 extracted premolars were randomly allocated to the experimental subgroups using a computer-generated randomization sequence (Random.org), ensuring balanced distribution across conditioning methods and adhesive types. All specimens were labeled with numerical codes that did not reveal group assignment. The operator who performed the SBS testing on the universal testing machine was blinded to the allocation of each specimen in order to minimize measurement bias.

### Group division

The 80 premolars were randomly divided into two groups, each containing 40 teeth:

Group G1 (Acid etching): Teeth in this group were further subdivided into four subgroups of 10 premolars each, corresponding to the four light-curing resins tested: Orthocem (FGM Dental Group, Brazil), Bracespace (3M, USA), Transbond XT (3M, USA), and Z250 (3M ESPE, USA) (Table 1). The enamel surfaces were conditioned using 37% phosphoric acid (Condac 37) for 15 seconds, followed by thorough rinsing with running water for 15 seconds and air drying until a chalky white surface was observed.

**Table 1:** Description of the four light-curing resins used in the study, including classification and composition.

Resin	Type of material	Classification	Main components/composition	Lot/batch number	Manufacturer’s instructions followed
Orthocem (FGM)	Orthodontic adhesive resin	Light-cured composite	Bis-GMA, TEGDMA, silica fillers	031126	No/Modified
Bracespace (3M)	Orthodontic adhesive resin	Nano-hybrid light-cured adhesive	Bis-GMA, UDMA, TEGDMA, silanated ceramic nanofillers	2422700664	No/Modified
Transbond XT (3M)	Orthodontic adhesive resin	Bis-GMA based light-cured composite	Bis-GMA, TEGDMA, ~70–80% silica fillers	2313800975	Yes
Z250 (3M)	Restorative micro-hybrid composite	Light-cured micro-hybrid composite resin	Bis-GMA, UDMA, Bis-EMA, zirconia/silica fillers (~60% vol.)	1370ª1	No/Modified

Bis-GMA: Bisphenol A glycidyl methacrylate; TEGDMA: Triethylene glycol dimethacrylate; UDMA: Urethane dimethacrylate; Bis-EMA: Ethoxylated bisphenol A dimethacrylate

Group G2 (Sandblasted bracket bases): Teeth in this group were also divided into four subgroups of 10 premolars each, using the same four resins as Group G1. However, in this group, the bracket bases were conditioned using a sandblasting technique. This involved a Bio-Art air abrasion device (Bio-Art, Brazil) connected to a pneumatic dental unit, operating at an air pressure of 60–80 psi (4–5.5 kgf/cm²). The sandblaster was positioned at a 138-degree angle, 10 mm from the bracket base, and the blasting process was done using 50-micron aluminum oxide particles for 15 seconds. Residual particles were removed by thorough water rinsing. These settings (50-µm aluminum oxide, 10 mm distance, 15 s exposure, and 60–80 psi pressure) were selected based on established methodologies in orthodontic adhesion research. Dirie et al. [17] used comparable abrasive conditions optimizing micromechanical retention, and Alvizo [5] demonstrated that 50-µm aluminum oxide particles at similar working distances produce reliable surface conditioning outcomes. The enamel surfaces in G2 were conditioned in the same way as G1, using 37% phosphoric acid, prior to bonding.

### Bracket debonding and surface preparation

**Figure 2:**
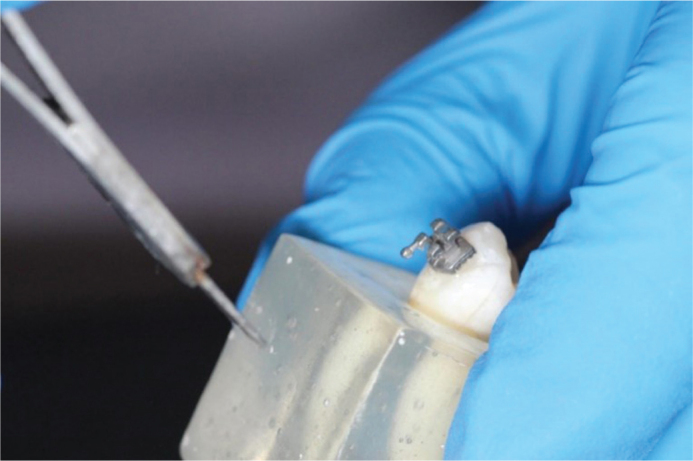
Bracket removal.

After the storage period, brackets were removed using angled bracket removing pliers (FALCON, Quinelato, Brazil) (Figure 2). Residual adhesive was removed using a #7901 multi-fluted tungsten carbide bur mounted on a high-speed handpiece operating at approximately 300,000 rpm, under continuous air cooling. Adhesive removal for each tooth required 20–30 seconds, depending on the amount of remaining composite. All procedures were performed by a single calibrated operator with more than 5 years of orthodontic bonding experience in order to minimize variability. The enamel surface was inspected under 2.5× loupe magnification to ensure complete removal of adhesive remnants while avoiding excessive enamel reduction.

To ensure proper enamel preparation, the tooth surfaces were marked with a pencil to identify the enamel regions and then polished with multi-fluted burs to create a clean bonding surface.

### Reconditioning process

Teeth in both the groups were reconditioned with 37% phosphoric acid.

The difference between the groups lies exclusively in the treatment of bracket bases:

G1: Bracket bases received no additional surface treatment.

G2: Bracket bases were sandblasted with 50-µm aluminum oxide particles before rebonding.

### Bracket bonding procedure

**Figure 3:**
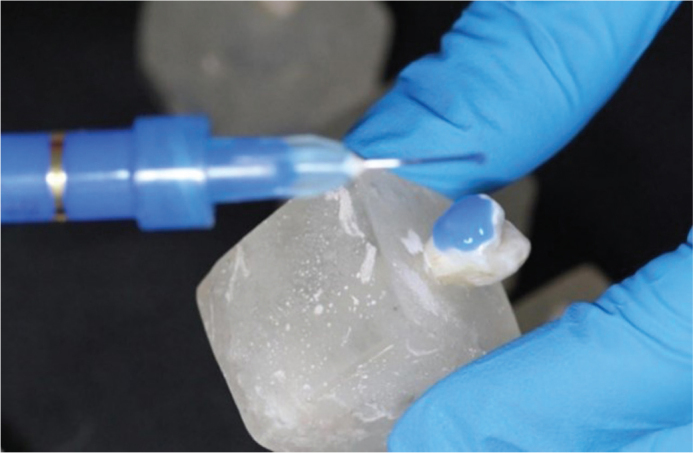
Acid etching of the enamel surface.

**Figure 4:**
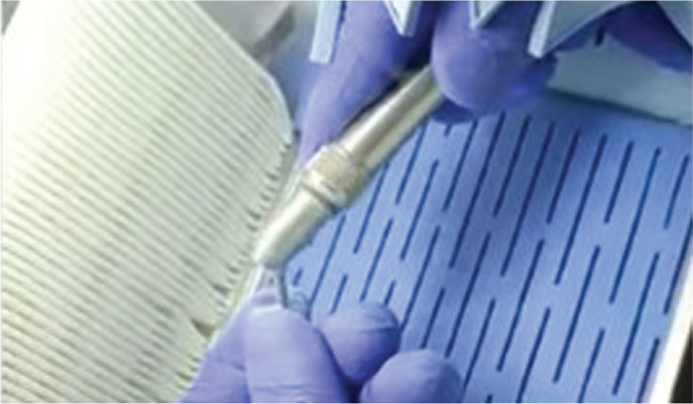
Sandblasting of bracket bases.

Before enamel etching, all premolars underwent deproteinization using 5.25% sodium hypochlorite (NaOCl) for 60 seconds with a prophylactic brush, following protocols reported in previous studies demonstrating that using NaOCl at concentrations of 5–6% for 30–60 seconds effectively removes organic debris, increases enamel surface energy, and enhances the penetration of phosphoric acid during etching. This step was performed prior to acid etching in both G1 and G2 to standardize enamel preparation (Figure 3), regardless of whether the bracket bases were sandblasted (Figure 4).

After this, the enamel surfaces of both the groups were etched with 37% phosphoric acid (Condac 37, FGM Dental Group, Brazil) for 15 seconds, followed by rinsing with running water for 15 seconds and air drying until a chalky white appearance was observed.

**Figure 5:**
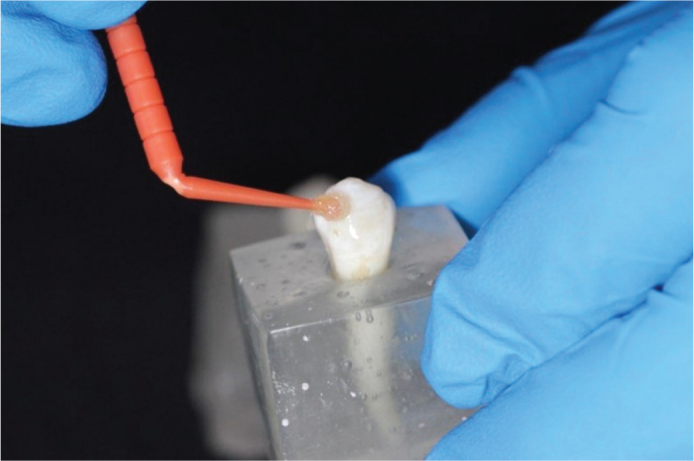
Adhesive application.

**Figure 6:**
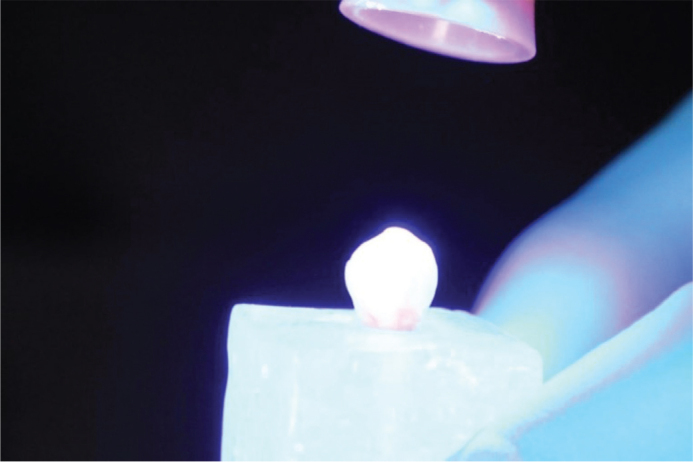
Light curing of adhesive.

Each tooth received a thin layer of Transbond XT light-cure adhesive (3M, USA) applied with a microbrush (Figure 5). The adhesive was light-cured for 3 seconds using a Woodpecker orthodontic curing light (Guilin Woodpecker Medical Instrument Co., Ltd., Guangxi, China) (Figure 6). The irradiance of the device was 1800–2000 mW/cm², as measured by the unit’s built-in radiometer, and the emitted wavelength ranged from 440 to 480 nm, according to the manufacturer’s specifications. These parameters were checked immediately before testing to ensure consistent light output throughout the experiment.

It should be noted that Transbond XT was applied exclusively as the adhesive layer (primer) on the enamel surface for all specimens, and not as the primary bonding material under investigation. The four experimental resins (Orthocem, Bracespace, Transbond XT, and Z250) were used only for bracket rebonding, maintaining the comparative integrity of the study.

**Figure 7:**
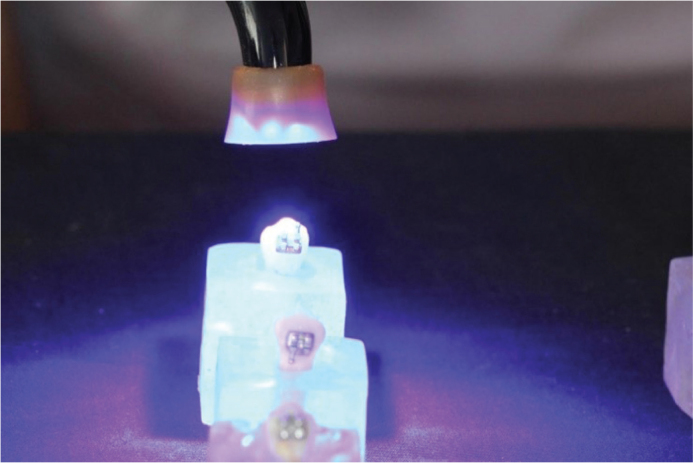
Light curing of resin after bracket placement.

Metal brackets (Roth 0.018” × 0.022”, Morelli, Brazil) were bonded to the enamel surface of each premolar. Excess resin around the brackets was carefully removed with a dental explorer, and the adhesive was cured for an additional 3 seconds using the same Woodpecker curing light (Figure 7).

Summarizing the sequence of conditioning steps: In both G1 and G2, deproteinization with 5.25% NaOCl was performed first, followed by etching using 37% phosphoric acid. In G2 only, sandblasting of the bracket bases was carried out before the bonding procedure. Thus, the order of procedures was: (1) enamel deproteinization, (2) enamel etching, (3) base conditioning (sandblasting only in G2), and (4) bracket rebonding.

It is important to clarify that Transbond XT was not tested as a separate bonding resin in place of the experimental materials. Instead, a very thin primer layer of Transbond XT was applied uniformly to the enamel surface in all specimens solely to standardize surface wetting and resin penetration. The actual bonding layer between the enamel and bracket base consisted of the experimental resins (Orthocem, Bracespace, Transbond XT, or Z250), which served as the independent variables in this study.

## Storage conditions between bonding cycles

After the initial bonding, all specimens were stored in physiological saline solution at room temperature (20–25°C) for 7 days before bracket debonding. Following adhesive removal and enamel surface reconditioning, the rebonded specimens were again stored under the same conditions (physiological saline, 20–25°C) for 7 days prior to shear testing.

No thermocycling or additional aging protocol was applied. The absence of artificial aging is acknowledged as a limitation, as it may restrict the extrapolation of results to long-term intraoral conditions.

Specimens were stored in physiological saline at 10–25°C, a condition widely used in SBS in-vitro models to prevent enamel dehydration without inducing hydrothermal degradation [20]. Storage at 37°C could accelerate resin water sorption and hydrolytic breakdown, artificially altering adhesive properties. Because our study evaluated short-term rebonding performance rather than long-term intraoral aging, the chosen temperature range is appropriate and avoids confounding variables.

## SBS testing

**Figure 8:**
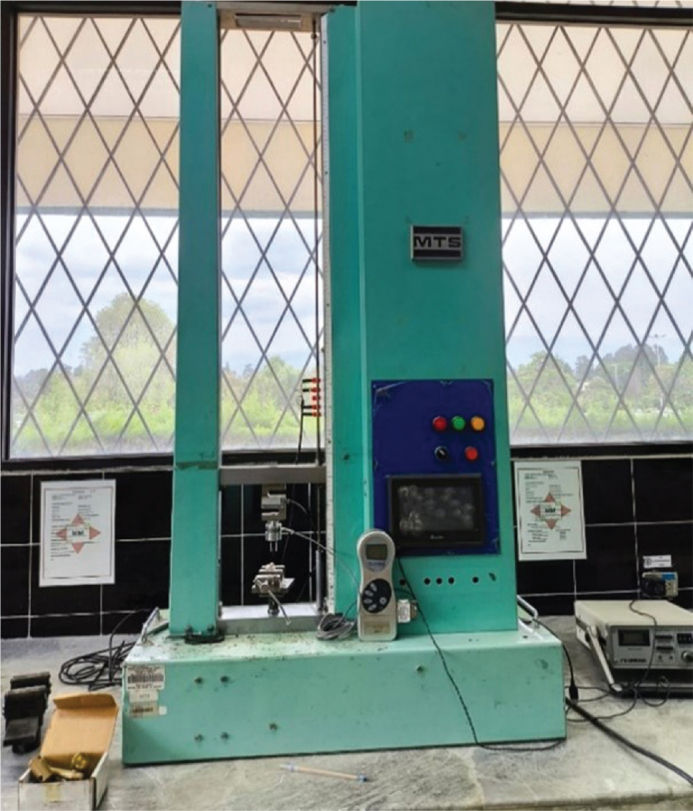
Universal testing machine.

**Figure 9:**
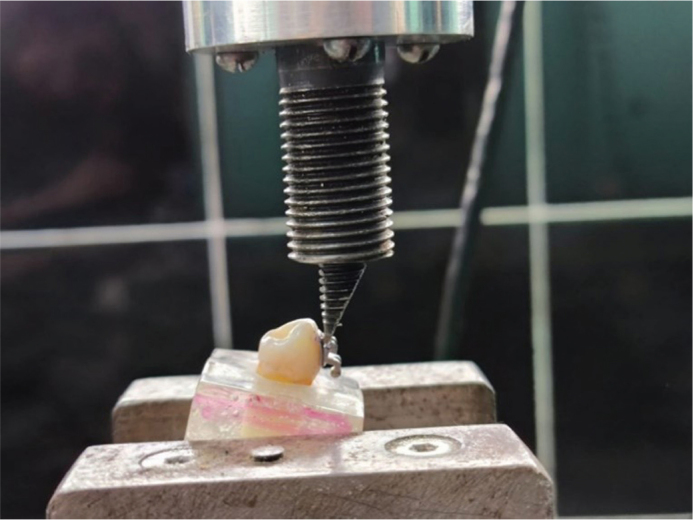
Shear bond strength testing.

SBS testing was performed using a universal testing machine (Model LLB400 1KLB ID 125014, Universidad Espe, Quito, Ecuador) at a crosshead speed of 0.5 mm/min (Figures 8 and 9). The universal testing machine was calibrated immediately prior to testing according to the manufacturer’s specifications. Calibration was verified using certified load cells traceable to international metrology standards, ensuring accuracy of force measurements during all SBS tests.

The force required to debond each bracket was recorded in kilograms-force (kgf) and later converted to megapascals (MPa) by dividing the force (in Newtons) by the area of the bracket base (4.97 mm²), following the relationship 1 *N*/mm² = 1 MPa.

The base area of the metal brackets used in this study was 4.97 mm², according to the manufacturer (Morelli, Brazil). This value was confirmed by measuring the mesiodistal and occlusogingival dimensions of the bracket base using a digital caliper (accuracy ± 0.01 mm).

The surface area was calculated using the formula: Area (mm^2^) = width (mm) × height (mm)

The measured values were consistent with the manufacturer’s reported base area. SBS was then calculated by dividing the debonding force (N) by the bracket base area (mm²) and converting the result to megapascals (MPa):

SBS (MPa) = Force (*N*)

Area (mm^2^)

The universal testing machine was calibrated according to the manufacturer’s specifications before testing. Load-cell precision was verified using traceable reference weights certified to international metrology standards (ISO 7500-1), ensuring accurate force readings.

**Figure 10:**
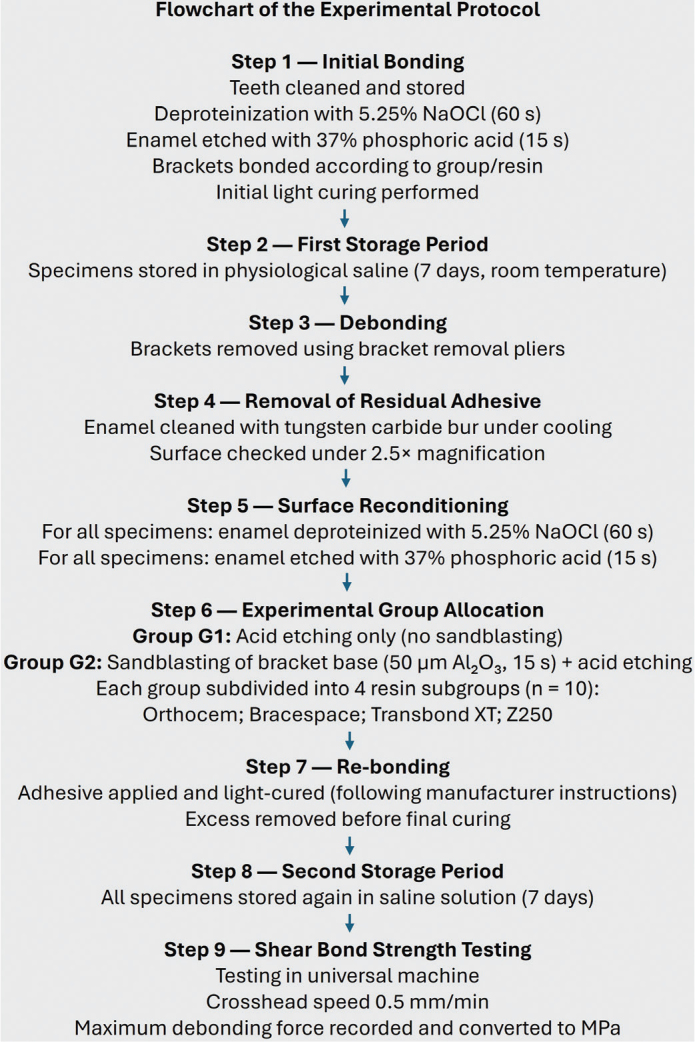
Flowchart of the experimental protocol.

For clarity and reproducibility, a graphical flowchart summarizing all experimental steps, from initial bonding through reconditioning and shear testing, is presented in Figure 10.

### Statistical analysis

The data were analyzed using SPSS (version 24) and Minitab, including normality testing (Shapiro-Wilk) and comparisons between groups using Kruskal-Wallis tests, with Dunn’s post-hoc test. Confidence intervals were set at 95%, and the data were initially collected in Newtons before conversion to MPa for final analysis.

## Results

For the statistical analysis of SBS, the Shapiro-Wilk test for normality found that the data did not follow a normal distribution. For this reason, the results were analyzed by the non-parametric Kruskal-Wallis test followed by Dunn’s post-hoc test. No outliers were identified according to the interquartile range criterion, and all values were retained for statistical analysis.

**Figure 11:**
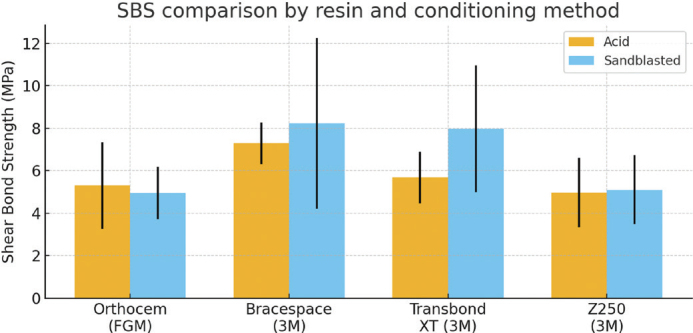
Comparison of mean shear bond strength (SBS) values obtained with acid etching and sandblasting conditioning protocols for the four light-cured resins tested. Error bars represent standard deviation (SD).

The results are presented in Table 2 and Figure 11. Whereas no significant differences were found among the four light-curing resins when the enamel had been acid-etched (*p* = 0.097), significant differences were found following sandblasting of bracket bases (*p* = 0.044). Post-hoc analysis found the SBS of Bracespace and Transbond to be significantly higher than those of Orthocem and Z250 (Table 2).

**Table 2:** Intergroup comparison (Kruskal-Wallis test).

Type	Resin	*N*	Mean	SD	95% CI		*p*
					Lower	Upper	
Acid	Orthocem (FGM)	10	5.30	2.04	2.76	7.84	0.097
	Bracespace (3M)	10	7.28	0.99	6.05	8.51	
	Transbond XT (3M)	10	5.68	1.22	4.15	7.20	
	Z250 (3M)	10	4.96	1.63	2.93	6.99	
Sandblasting	Orthocem (FGM)	10	4.94 A	1.23	3.41	6.47	0.044*
	Bracespace (3M)	10	8.23 B	4.02	3.23	13.22	
	Transbond XT (3M)	10	7.98 B	2.98	4.27	11.68	
	Z250 (3M)	10	5.10 A	1.61	3.09	7.11	

Different letters indicate the presence of a statistically significant difference indicated by the Dunn’s post-hoc test.

## Discussion

The primary objective of this study was to compare the SBS of rebonded brackets using four different light-curing resins under two conditioning protocols: acid etching of enamel and sandblasting of bracket bases. The results showed that, in the acid-etched group, no significant differences were found among the four resins (*p* = 0.097), indicating comparable SBS regardless of the adhesive. In contrast, when the bracket bases were sandblasted, a significant intergroup difference was observed (*p* = 0.044). Dunn’s post-hoc test revealed that Bracespace and Transbond XT produced significantly higher SBS than Orthocem and Z250. These findings demonstrate that the resin type influenced bond strength only under the sandblasting condition, whereas acid etching alone did not differentiate the performance of the adhesives.

In terms of clinical relevance, Reynolds proposed that an SBS range of approximately 5.9–7.8 MPa is sufficient to withstand masticatory forces while minimizing the risk of enamel damage during debonding [21]. Based on this benchmark, the mean SBS values obtained for Bracespace and Transbond XT under sandblasting (8.23 and 7.98 MPa, respectively) are at or slightly above the upper limit of the clinically acceptable range, whereas most of the other subgroups lie close to, or slightly below, its lower threshold. In particular, Orthocem (4.94–5.30 MPa) and Z250 (4.96–5.10 MPa) showed mean values that are marginally lower than 6 MPa, which may indicate a greater risk of bracket failure under high functional loading compared with the resins that consistently reached or exceeded the proposed range. Nonetheless, these values are not dramatically lower than the suggested clinical threshold, and they still represent bond strengths that could be acceptable in low-load clinical situations; however, from a practical standpoint, resins that reliably achieve SBS values around or above 6–7 MPa are preferable for routine orthodontic bonding.

Consistent with the findings of Alvizo [5], the present study showed that sandblasting of the bracket bases improved bond strength only for specific resins. A significant increase in SBS was observed for Bracespace and Transbond XT when compared with their respective acid-etched conditions, whereas Orthocem and Z250 did not exhibit statistically significant differences between conditioning methods. This indicates that the effect of sandblasting is resin-dependent, rather than a universal improvement applicable to all materials, and reinforces the importance of selecting conditioning protocols according to the adhesive system used. These findings are in agreement with the observations of Basudan and Al-Emran [6] regarding the selective benefits of sandblasting on mechanical retention.

The differential response to sandblasting is likely related to intrinsic physicochemical properties of the adhesive systems. Bracespace and Transbond XT possess intermediate-to-high viscosity and high filler content, which may allow more effective penetration and mechanical interlocking into the micro-retentive pattern created by airborne particle abrasion. Their matrix composition (Bisphenol A glycidyl methacrylate-based (bis-GMA) formulations with balanced diluent monomers) supports adequate wettability and continuation of flow after contact with the roughened metallic base. In contrast, Orthocem and Z250 exhibit lower viscosity and comparatively reduced capacity to fill micro-irregularities generated by sandblasting, which may limit the extent of micromechanical interlocking. Additionally, Z250 contains larger filler particles intended primarily for restorative applications, which may hinder intimate adaptation to the sandblasted surface. Therefore, the improvement in SBS observed only with Bracespace and Transbond XT can be attributed to improved wetting behavior, filler distribution, and polymer matrix characteristics that more effectively match the microroughness generated by sandblasting.

However, it is important to note that other studies, such as Urichianu et al. [22] found that sandblasting can damage the retentive features of ceramic bracket bases, leading to lower SBS values in certain brackets like Symetri Clear and Radiance Plus. This difference may be attributed to the physical properties of ceramic brackets, which differ significantly from metal brackets. In contrast, the present study focused exclusively on metal brackets, which may better withstand the abrasive effects of sandblasting, particularly when combined with the use of NaOCl for deproteinization, as described in the methodology.

The use of NaOCl in this study likely contributed to the enhanced bond strength observed, as it effectively removes organic debris and residual proteins from the enamel surface, improving the retention of adhesives. This approach is supported by studies demonstrating that enamel deproteinization with NaOCl can significantly improve bonding strength by increasing the surface energy and creating a more receptive bonding substrate [23, 24]. Al Daher et al. [23] further demonstrated that NaOCl may produce comparable improvements to sandblasting in rebonding protocols, supporting its application in the present methodology.

The use of NaOCl in this study likely contributed to the enhanced bond strength observed, as it effectively removes organic debris and residual proteins from the enamel surface, improving the retention of adhesives. This approach is supported by studies demonstrating that enamel deproteinization with NaOCl can significantly improve bonding strength by increasing the surface energy and creating a more receptive bonding substrate [23, 24]. Notably, Al Daher et al. [23] compared enamel reconditioning achieved by NaOCl deproteinization versus sandblasting and concluded that both methods improve adhesion, although sandblasting may provide superior micromechanical retention when extensive rebonding is required. This supports the rationale for combining deproteinization with mechanical preparation, as adopted in the present study.

However, evidence in the literature remains inconclusive regarding the overall impact of NaOCl on SBS. Recent systematic reviews and experimental investigations have reported no statistically significant gain in SBS or Adhesive Remnant Index (ARI) following NaOCl pretreatment [25, 26]. Therefore, the improved bond performance observed in some cases cannot be unequivocally attributed to NaOCl.

Interestingly, the findings also partially align with those of Sharma et al. [27], who reported significantly higher SBS for brackets bonded with Transbond XT (15.49 MPa), although this study observed lower SBS values for the same resin (7.98 MPa with sandblasting and 5.68 MPa with acid conditioning). This discrepancy could be attributed to differences in experimental design, including the type of brackets, adhesive application techniques, and test methodologies. A likely explanation for the markedly higher SBS reported by Sharma et al. [27] lies in methodological differences between studies. They used ceramic brackets with a different base design, whereas the present investigation employed metal brackets, which exhibit distinct mechanical retention characteristics. Additionally, their bonding protocol involved a longer light-curing time and potentially higher irradiance output, both of which can enhance polymerization and thereby increase SBS. Differences in storage media and thermocycling may also have contributed, as no aging protocol was applied in the present study. These procedural variations provide a rational justification for the numerical discrepancy between results.

The current results also highlight the potential influence of resin viscosity and handling characteristics on SBS. Bracespace, which has an intermediate viscosity, showed significantly higher SBS values than Orthocem and Z250 only under the sandblasting condition, suggesting that improved mechanical interlock of the bracket base may benefit certain adhesive formulations. However, in the acid-etched group, no significant differences were detected among the four resins, indicating that Orthocem and Z250 did not perform worse than the other materials under this conditioning method. These findings support the notion that the effect of viscosity on SBS is resin-dependent and manifests only under specific surface-conditioning conditions, consistent with the observations of Al-Saleh and El-Mowafy [28].

Based on the findings, this hypothesis was partially supported, since sandblasting increased SBS only for Bracespace and Transbond XT, while Orthocem and Z250 did not show significant improvement.

This study presents several limitations that should be considered when interpreting the results. First, as an in vitro experiment, the bonding and debonding processes were performed under controlled laboratory conditions that do not reproduce the variability present in the oral cavity, including salivary contamination, masticatory forces, pH fluctuations, and long-term thermal changes. Second, specimens were stored for relatively short periods, and no thermocycling or cyclic fatigue loading was applied; these aging conditions may alter adhesive performance over time and are relevant to clinical reality. Additionally, only a single rebonding cycle was simulated, whereas patients may undergo multiple bracket replacements during treatment, potentially producing cumulative changes in enamel morphology and adhesion. The experimental design evaluated four commercially available resins, but did not encompass the full range of orthodontic adhesives, limiting generalizability. The absence of ARI analysis limits the understanding of failure mode patterns and interface characterization in this study. Finally, SBS testing based on a unidirectional shear load does not fully simulate the complex 3D functional forces acting on orthodontic brackets intraorally. Taken together, these limitations reinforce the need for complementary in vivo studies and long-term durability assessments to validate the clinical applicability of the present findings.

## Conclusions

The findings of this in vitro study demonstrated that, after enamel acid etching, no statistically significant differences in SBS were detected among the four light-curing resins evaluated. In contrast, when bracket bases were sandblasted, significant differences emerged: Bracespace and Transbond XT achieved higher SBS values than Orthocem and Z250, as confirmed by post-hoc analysis. Thus, the influence of surface conditioning was resin-dependent, with sandblasting improving bond strength only for Bracespace and Transbond XT, while Orthocem and Z250 showed no significant improvement under the same protocol. These results suggest that bracket-base surface reconditioning may play a key role in optimizing SBS for specific adhesive systems. However, all findings are limited to controlled laboratory conditions and require clinical validation before translation to in vivo orthodontic practice.

Based on these laboratory findings, clinicians performing bracket rebonding may achieve optimal in vitro SBS by using sandblasting (50-micron aluminum oxide at 60–80 psi) for bracket base preparation combined with either Bracespace or Transbond XT resins. However, prospective clinical studies are necessary to validate these results under actual intraoral conditions and to determine long-term bracket survival rates.

## Declaration of interest

The authors declare no competing interests.

## Authors contributions

Veronica Estefania Pozo - Conceptualization, Data curation, Investigation, Methodology, Data curation, Software, Validation, Writing original draft

Marjory Elizabeth Vaca Zapata – Conceptualization, Data curation, Investigation, Investigation, Writing – review and editing

Mauricio Aguirre Balseca – Formal analysis, Funding acquisition, Investigation, Visualization, Software, Writing – review and editing

Karina Maria Salvatore Freitas – Formal analysis, Methodology, Validation, Writing original draft, Writing – review and editing

Alex Dario Ganan - Conceptualization, Data curation, Investigation, Formal analysis, Methodology, Project administration; Resources; Supervision, Writing – review and editing

## Data availability

The datasets generated and/or analyzed during the current study are available from the corresponding author on reasonable request.

## Ethics approval

This study was approved by the Institutional Ethics Committee of the University of the Hemispheres, Quito, Ecuador, under protocol number CEUHE 24-48 (IRB number).

## Consent for publication

Not applicable.
